# Histone H3K9 Demethylase JMJD2B Activates Adipogenesis by Regulating H3K9 Methylation on PPARγ and C/EBPα during Adipogenesis

**DOI:** 10.1371/journal.pone.0168185

**Published:** 2017-01-06

**Authors:** Min-Kyung Jang, Ji-Hyun Kim, Myeong Ho Jung

**Affiliations:** School of Korean Medicine, Pusan National University, 49 Busandaehak-ro, Mulguem-eup, Yangsan-si, Gyeongnam, South Korea; University of Minnesota Twin Cities, UNITED STATES

## Abstract

Previous studies have shown that tri- or di-methylation of histone H3 at lysine 9 (H3K9me3/me2) on the promoter of the peroxisome proliferator-activated receptor γ (PPARγ) and CCAAT/enhancer-binding protein α (C/EBPα) contribute to the repression of PPARγ and C/EBPα and inhibition of adipogenesis in 3T3-L1 preadipocytes. The balance of histone methylation is regulated by histone methyltransferases and demethylases. However, it is poorly understood which demethylases are responsible for removing H3K9me3/me2 on the promoter of PPARγ and C/EBPα. JMJD2B is a H3K9me3/me2 demethylase that was previously shown to activate adipogenesis by promoting mitotic clonal expansion. Nevertheless, it remains unclear whether JMJD2B plays a role in the regulation of adipogenesis by removing H3K9me3/me2 on the promoter of PPARγ and C/EBPα and subsequently activating PPARγ and C/EBPα expression. Here, we showed that JMJD2B decreased H3K9me3/me2 on the promoter of PPARγ and C/EBPα, which in turn stimulated the expression of PPARγ and C/EBPα. JMJD2B knockdown using siRNA in 3T3-L1 preadipocytes repressed the expression of PPARγ and C/EBPα, resulting in inhibition of adipogenesis. This was accompanied by increased enrichment of H3K9me3/me2 on the promoter of PPARγ and C/EBPα. In contrast, overexpression of JMJD2B increased the expression of PPARγ and C/EBPα, which was accompanied by decreased enrichment of H3K9me3/me2 on the promoter and activated adipogenesis. Together, these results indicate that JMJD2B regulates PPARγ and C/EBPα during adipogenesis.

## Introduction

The differentiation of preadipocytes into adipocytes (adipogenesis) is modulated by diverse transcription factors that coordinate the expression of genes responsible for determining the mature fat-cell feature [1, 2]. At the early stage of adipogenesis, the transcription factors, including the CCAAT/enhancer-binding protein (C/EBP) β/δ, glucocorticoid receptor (GR), Krüppel-like factor 5 (KLF5), cAMP response element-binding protein (CREB), early growth response protein 2 (EGR2 or Krox20), and sterol regulatory element-binding protein 1c (SREBP-1c) are induced, thereby stimulating the expression of the peroxisome proliferator-activated receptor γ (PPARγ) and C/EBPα. The key adipogenic factors, PPARγ and C/EBPα, in turn stimulate the expression of the genes for the mature adipocyte phenotype, including adipocyte fatty acid-binding protein (aP2), CD36, and adiponectin. In addition, PPARγ and C/EBPα reciprocally stimulate each other to mediate the transition of preadipocyte to the adipocyte phenotype. In contrast, the Wnt/β-catenin pathway serves as negative regulator of adipocyte differentiation.

The methylation of lysine residues in histones is a main epigenetic modification in the regulation of eukaryotic gene expression. While methylation of histone H3 at the lysine 4 (H3K4) and 36 (H3K36) residues primarily associates with active transcription, methylation of histone H3 at the lysine 9 (H3K9) and 27 (H3K27) residues and histone H4 at lysine 20 (H4K20) associates with gene repression. Specific histone methyltransferases or demethylases are involved in methylation and demethylation at each amino acid position. It has been reported that several histone methyltransferases regulate adipogenesis by modulating the expression of PPARγ and C/EBPα. H3K9 methyltransferases, including Setdb1 [3], G9a [4], and Suv39h1 [5], negatively regulate adipogenesis through di- or tri-methylation of histone H3 at lysine 9 (H3K9me2/me3) on PPARγ and C/EBPα. Setdb1 increases H3K9me3 on PPARγ and inhibits PPARγ expression, and Suv39h1 increases H3K9me3 on both PPARγ and C/EBPα and represses the expression of PPARγ and C/EBPα [3, 5]. G9a also inhibits PPARγ expression by increasing H3K9me2 on PPARγ [4]. In contrast, the H4K20 monomethyltransferase PR-Set7/Setd8 positively stimulates adipogenesis by increasing H4K20 monomethylation (H4K20me) on PPARγ, thus activating the expression of PPARγ and its targets [6]. The H3K27 methyltransferase enhancer of zeste homolog 2 (EZH2) induces adipogenesis by silencing genes involved in the Wnt pathway [7].

Histone methylation by histone methyltransferase is antagonized by histone demethylases, which are divided into two classes: amine oxidases (LSD demethylases), and jumonji C (JmjC) domain-containing, iron-dependent dioxygenases (JMJC demethylases) [8]. JmjC domain-containing histone demethylases are further classified into several subfamilies based on substrate specificity for H3K4, H3K9, H3K27 or H3K36 [9]. The demethylation state of H3K9 is influenced by LSD1 and several JMJC demethylases. While LSD1 demethylates mono- and di-methylated lysine 4 and lysine 9 on histone 3 [10, 11], JMJD2 (also known as KDM4) family including JMJD2A (KDM4A), JMJD2B (KDM4B) and JMJD2C (KDM4C) catalyzes the removal of di- and tri-methylated lysine 9 and lysine 36 on histone H3 [12, 13]. Previous study showed that LSD1 demethylates H3K9me2 at the C/EBPα promoter region and promotes adipocyte differentiation [14]. However, histone demethylases that erase H3K9me3 or H3K9me2 on the promoter regions of PPARγ and C/EBPα have not been well characterized. Very recently, a study showed that JMJD2B demethylates H3K9me3 on C/EBPβ target genes and promote mitotic clonal expansion, resulting in stimulation of adipogenesis [15]. However, it is still unknown whether JMJD2B mediates the enrichment of H3K9me3/me2 on PPARγ and C/EBPα, thus activating the expression of PPARγ and C/EBPα.

Here, we show that JMJD2B reduces the enrichment of H3K9me3/me2 on the promoters of PPARγ and C/EBPα and stimulates PPARγ and C/EBPα expression, which may play an important role in the promotion of terminal adipogenesis in 3T3-L1 preadipocytes.

## Materials and Methods

### Cell culture and differentiation of 3T3-L1

The 3T3-L1 preadipocytes used in this study were obtained from the American Type Culture Collection (Manassas, VA, USA) and were cultured in Dulbecco’s modified Eagle’s medium (DMEM) containing 10% fetal bovine serum (FBS), and 1% penicillin/streptomycin. To differentiate the 3T3-L1 preadipocytes, 90% confluent preadipocytes at day 0 were incubated in a differentiation medium containing 20 nM insulin, 1 nM T3, 125 μM indomethacin, 500 μM isobutylmethylxanthine (IBMX), and 0.5 μM dexamethasone for 2 days, and then treated with the differentiation medium supplemented with 20 nM insulin and 1 nM T3. Insulin, T3, indomethacin, IBMX, and dexamethasone were purchased from Sigma Aldrich (St. Louis, MO, USA). DMEM, FBS, and penicillin/streptomycin were purchased from Life Technologies (Grand Island, NY, USA)

### Oil Red O staining

The 3T3-L1 adipocytes were fixed overnight with 4% paraformaldehyde, washed with 60% isopropyl alcohol, and stained with Oil Red O solution (0.21% Oil Red O in 60% isopropyl alcohol) for 1 h at room temperature. Cells were washed with water and photographed.

### Transfection of 3T3-L1 cells

To deplete JMJD2B, duplex of siRNA targeting JMJD2B (sense: 5’-CCAGUUCAGUAUCAAUUAAAGCCCG-3’, antisense: 5’-CGGGCUUUAAUUGAUACUGAACUGGAG-3’) was designed and synthesized by Integrated DNA Technologies (Coralville, Iowa, USA). The JMJD2B expression vector (pCMV-JMJD2B) was purchased from Addgene (Cambridge, MA, USA). The 3T3-L1 preadipocytes were transfected with the siRNAs or pCMV-JMJD2B using the lipofectamine RNAiMAX reagent kit or lipofectamine LTX with PLUS reagent kit (Invitrogen, Carlsbad, CA, USA). The transfected cells were differentiated in the differentiation medium 6 days after transfection.

### Total RNA preparation and quantitative real-time PCR (qPCR)

Total RNA was extracted using TRIZOL® (Invitrogen) according to the manufacturer's instructions. The cDNA was generated from 1 μg of total RNA using the GoScript™ Reverse Transcription System (Promega, Madison, WI, USA) according to the manufacturer's protocol. PCR amplification was performed using gene specific primers. The primers used in this study are listed in supplemental data, S1 Table.

### Western blot

Equal amounts of protein (40 μg/lane) from the 3T3-L1 cell lysates were resolved by 8% SDS-polyacrylamide gel electrophoresis (SDS-PAGE), transferred to polyvinylidene difluoride membranes (Millipore, Billerica, MA, USA), and immunoblotted with antibodies against JMJD2B, PPARγ, C/EBPα, H3K9me2, H3K9me3, and histone H3. The antibodies for JMJD2B (#ab91549), PPARγ (#ab41928), C/EBPα (#ab15048) were purchased from Abcam (Cambridge, MA, USA). Antibodies for H3K9me2 (#07–441), H3K9me3 (#07–442) and H3 (#06–755) were purchased from Millipore. The proteins were detected using an enhanced chemiluminescence western blot detection kit (Amersham, Uppsala, Sweden).

### Chromatin immunoprecipitation (ChIP)-qPCR

ChIP was performed as described [16]. Briefly, the 3T3‐L1 preadipocytes or 3T3-L1 adipocyte cells were fixed with 1% formaldehyde for 10 min at room temperature. The crosslinked chromatins were sonicated to shear to 400 bp fragments using a Bioruptor sonicator (Diagenode, Denville, NJ, USA). Samples were immunoprecipitated using 1–2 μg antibodies against H3K9me2, H3K9me3 and H3K4me3 (Millipore) or non-specific IgG control (Abcam) in the presence of secondary antibody conjugated to Dynabeads (Invitrogen). Purified DNA was subjected to qPCR using the the following primers: PPARγ sense: 5′-CCCTCACAGAACAGTGAATGTGT-3′; PPARγ antisense: 5′- TGCTTTGGCAAGACTTGGTACAT-3′; C/EBPα sense: 5′- CCACTCACCGCCTTGGAA-3′; and C/EBPα antisense: 5′-GTCCAAACGGGTCTCGGA-3′. ChIP data were normalized to control IgG or expressed as a percentage of input.

### Statistical analysis

Data are expressed as the mean ± SEM. Statistically significant differences were determined by the two-tailed Student's t-test. For all statistical analyses, *p* values below 0.05 were considered significant.

## Results

### Expression of JMJD2D is correlated with that of PPARγ and C/EBPα during adipogenesis

**Fig 1:**
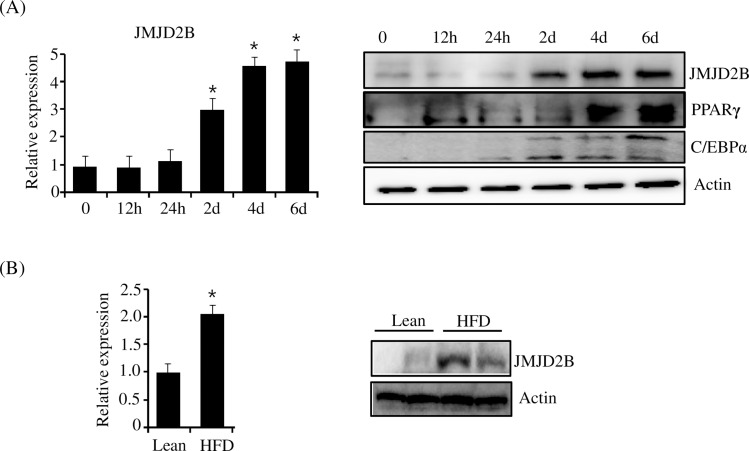
Expression of JMJD2B increased during differentiation of 3T3-L1 preadipocytes and in the white adipose tissue of HFD mice. (A) The 3T3-L1 preadipocytes were differentiated in the differentiation medium. At the indicated time, the expression of JMJD2B was measured by qPCR. Protein levels for JMJD2B, PPARγ, and C/EBPα were also assessed by western blot. Data are presented as means±SEM from three independent experiments. *P< 0.05 vs. 3T3-L1 preadipocyte (0day). (B) Total RNAs were prepared from adipose tissues of HFD obese mice. The expression of JMJD2B was measured by qPCR (left). Data are presented as means±SEM from three independent experiments. *P< 0.05 vs. Lean. Tissue lysates prepared from adipose tissues of HFD obese mice were subjected to western blot using JMJD2B antibody (right)

To determine whether the expression of JMJD2D is correlated with that of PPARγ and C/EBPα during adipogenesis, we examined the expressions of JMJD2D, PPARγ, and C/EBPα at certain periods after induction of adipogenesis. As shown in Fig 1A, the expression of JMJD2B increased from day 2, which was similar with the expression profiles of PPARγ and C/EBPα. Western blot also showed similar expression patterns between JMJD2B and PPARγ or C/EBPα, suggesting that JMJD2B may stimulate PPARγ and C/EBPα expression during adipogenesis. Furthermore, we examined JMJD2B expression in the adipose tissue of high-fat diet (HFD) obese mice. The expression of JMJD2B was higher in the adipose tissue of HFD obese mice compared with that of lean mice, which was revealed by qPCR and western blot (Fig 1B).

### JMJD2B promotes adipogenesis in 3T3-L1 preadipocytes

**Fig 2:**
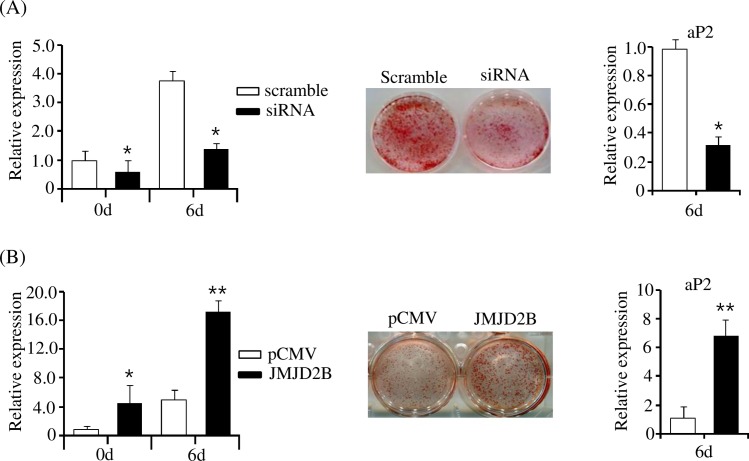
JMJD2B stimulates differentiation of 3T3-L1 preadipocytes. (A) Knockdown of JMJD2B decreased differentiation of 3T3-L1 preadipocytes. The 3T3-L1 preadipocytes were transfected with JMJD2B siRNA and differentiated in the differentiation medium for six days. The expression of JMJD2B was measured at days 0 (0d) and 6 (6d) by qPCR. The 3T3-L1 adipocyte cells were stained with Oil Red O. The expression of aP2 was measured by qPCR. Data are presented as means±SEM from three independent experiments. *P< 0.05 vs. scramble RNA. (B) Overexpression of JMJD2B increased differentiation of 3T3-L1 preadipocytes. The 3T3-L1 preadipocytes were transfected with the pCMV-JMJD2B and differentiated in the differentiation medium for six days. The expression of JMJD2B was measured at days 0 (0d) and 6 (6d) by qPCR. The 3T3-L1 adipocyte cells were stained with Oil Red O. The expression of aP2 was measured by qPCR. Data are presented as means±SEM from three independent experiments. **P< 0.01 vs. control vector pCMV.

To confirm the role of JMJD2B in adipogenesis, we examined the effect of JMJD2B knockdown in 3T3-L1 preadipocytes. The 3T3-L1 preadipocytes were treated with JMJD2B siRNA or scramble RNA and then stimulated to differentiate in a different medium for six days. To verify the knockdown of JMJD2B using siRNA, we determined the expression of JMJD2B in siRNA-transfected 3T3-L1 cells. The qPCR analysis showed that treatment with JMJD2B siRNA efficiently reduced JMJD2B expression in both 3T3-L1 preadipocyte cells at day 0 and differentiated adipocyte cells at day 6 (Fig 2A). Oil Red O staining showed JMJD2B knockdown inhibited adipogenesis of 3T3-L1 adipocyte cells, which was also revealed by the expression of the adipogenic gene aP2 (Fig 2A).

To further confirm the positive effect of JMJD2B on adipogenesis, we determined adipogenesis in JMJD2B-overexpressed 3T3-L1 adipocyte cells. The 3T3-L1 preadipocyte cells were transfected with the JMJD2B expression vector and differentiated for six days. qPCR showed that JMJD2B efficiently increased in 3T3-L1 preadipocytes transfected with the JMJD2B expression vector. After six days of differentiation, adipogenesis was stimulated in JMJD2B-overexpressed 3T3-L1 adipocyte cells compared with control vector-transfected adipocyte cells, which was revealed by Oil Red O staining and expression of aP2 (Fig 2B). Altogether, these results validate that JMJD2B promotes adipogenesis in 3T3-L1 cells.

### JMJD2B stimulates expression of PPARγ and C/EBPα

**Fig 3:**
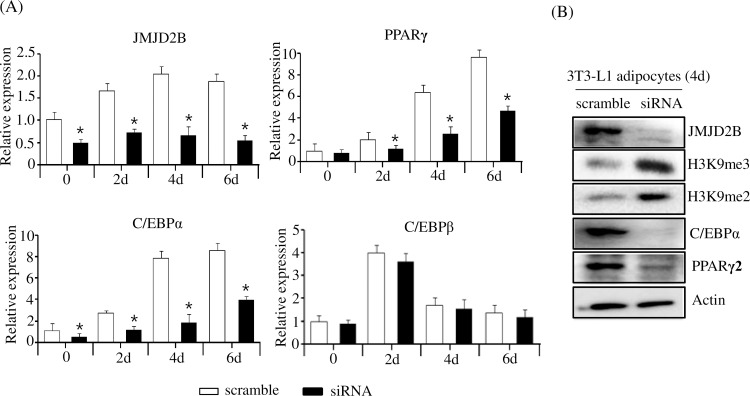
Knockdown of JMJD2B decreased expression of PPARγ and C/EBPα. The 3T3-L1 preadipocytes were transfected with JMJD2B siRNA or scramble RNA and differentiated in the differentiation medium for six days. (A) The expression of JMJD2B, PPARγ, C/EBPα, and C/EBPβ was measured at the indicated times by qPCR. Data are presented as means±SEM from three independent experiments. *P< 0.05 vs. scramble RNA. (B) Protein levels of JMJD2B, H3K9me3, H3K9me2, PPARγ, and C/EBPα were measured at day 4 by western blot.

Next, to identify the JMJD2B target genes, we examined the expression of adipogenic regulators in JMJD2B knockdown 3T3-L1 adipocyte cells. The 3T3-L1 preadipocytes were transfected with either siRNA or scramble RNA and stimulated to differentiate for six days. The expression of adipogenic regulators including PPARγ, C/EBPα, and C/EBPβ was determined at days 0, 2, 4, and 6. The qPCR analysis showed that the expression of JMJD2B efficiently decreased in the 3T3-L1 adipocyte cells at days 0, 2, 4, and 6 using siRNA. Concomitant with the decreased expression of JMJD2B, the expression of PPARγ and C/EBPα significantly decreased in JMJD2B siRNA-transfected 3T3-L1 cells at days 2, 4, and 6 compared with scramble RNA-transfected 3T3-L1 cells (Fig 3A). However, C/EBPβ expression was not affected by treatment with JMJD2B siRNA (Fig 3A). To validate these results, we measured the protein levels of PPARγ and C/EBPα from the extracts of JMJD2B siRNA-transfected 3T3-L1 cells at day 4 by western blot. Consistent with the result of qPCR, the JMJD2B protein level decreased in JMJD2B siRNA-transfected 3T3-L1 cells, thus increasing global H3K9me3 and H3K9me2 (Fig 3B). The protein levels of PPARγ and C/EBPα significantly decreased in siRNA-transfected 3T3-L1 adipocyte cells at day 4 (Fig 3B). These results suggest that JMJD2B stimulates PPARγ and C/EBPα expression during adipogenesis in 3T3-L1 preadipocytes.

**Fig 4:**
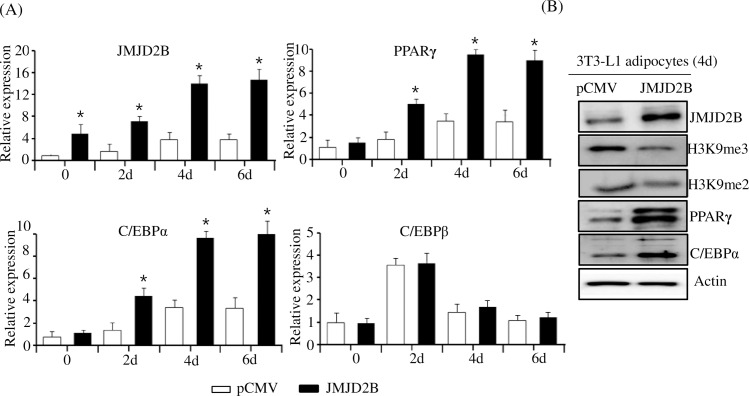
Overexpression of JMJD2B increased the expression of PPARγ and C/EBPα. The 3T3-L1 preadipocytes were transfected with JMJD2B expression vector and differentiated in the differentiation medium for four days. (A) The expression of JMJD2B, PPARγ, C/EBPα, and C/EBPβ was measured at the indicated times by qPCR. Data are presented as means±SEM from three independent experiments. *P< 0.05 vs. control vector pCMV. (B) The protein levels of JMJD2B, H3K9me3, H3K9me2, PPARγ, and C/EBPα were measured at day 4 by western blot.

To further confirm the positive effect of JMJD2B on PPARγ and C/EBPα expression, we examined the expression of PPARγ and C/EBPα in JMJD2B-overexpressed 3T3-L1 adipocyte cells. The 3T3-L1 preadipocytes were transfected with the JMJD2B expression vector and differentiated for four days. The qPCR analysis showed that the expression of JMJD2B significantly increased in JMJD2B expression vector-transfected 3T3-L1 cells at days 0, 2, 4 and 6 (Fig 4A). Concomitant with increased expression of JMJD2B, expression of PPARγ and C/EBPα greatly increased in JMJD2B expression vector-transfected 3T3-L1 cells compared with control vector-transfected 3T3-L1 cells (Fig 4A). Western blot also showed that the protein levels of JMJD2B, PPARγ, and C/EBPα increased in JMJD2B expression vector-transfected 3T3-L1 adipocyte cells (Fig 4B). In contrast, global H3K9me3 and H3K9me2 significantly decreased in JMJD2B-overexpressed 3T3-L1 cells (Fig 4B). These results indicate that JMJD2B upregulates PPARγ and C/EBPα during the adipogenesis of 3T3-L1.

### JMJD2B decreases H3K9me3/me2 levels on the promoters of PPARγ and C/EBPα

**Fig 5:**
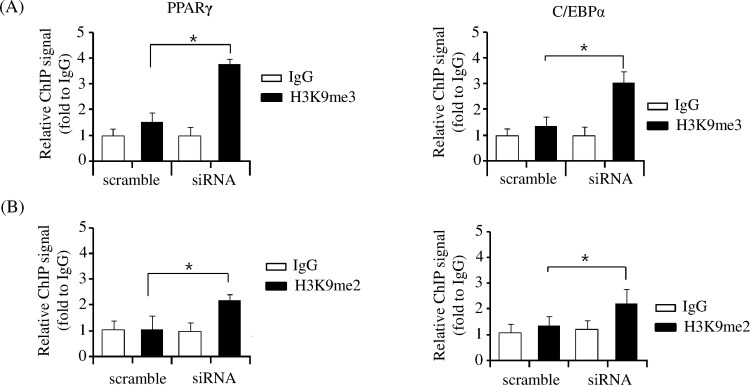
Knockdown of JMJD2B decreased the enrichment of H3K9me3 and H3K9me2 on the promoter of PPARγ and C/EBPα. The 3T3-L1 preadipocytes were transfected with JMJD2B siRNA and differentiated for four days. The enrichment of H3K9me3 (A) and H3K9me2 (B) on the promoters of PPARγ and C/EBPα was analyzed by ChIP-qPCR. Data are presented as means±SEM from three independent experiments. *P< 0.05 vs. scramble RNA.

Recent reports showed that both H3K9me3 and H3K9me2 on PPARγ and C/EBPα repressed PPARγ and C/EBPα expression [3–5]. H3K9me3 and H3K9me2 were enriched on the promoter of PPARγ and C/EBPα in the preadipocytes and decreased during differentiation. Since JMJD2B is a H3K9me3/me2 demethylase and based on the observations in the present study, also reduces global H3K9me3 and H3K9me2 in 3T3-L1 cells, we investigated whether JMJD2B regulates the enrichment of H3K9me3 and H3K9me2 on the promoters of PPARγ and C/EBPα. To this end, the 3T3-L1 preadipocytes were treated with scramble or JMJD2B siRNA and stimulated to differentiate for four days, and the enrichment of H3K9me3 and H3K9me2 on the promoters of PPARγ and C/EBPα was examined by ChIP assay. ChIP-qPCR showed that the enrichment of H3K9me3 on the promoters of PPARγ and C/EBPα increased in JMJD2B siRNA-transfected 3T3-L1 cells compared with scramble RNA-transfected cells (Fig 5A). Furthermore, the enrichment of H3K9me2 also increased by JMJD2B knockdown (Fig 5B).

**Fig 6:**
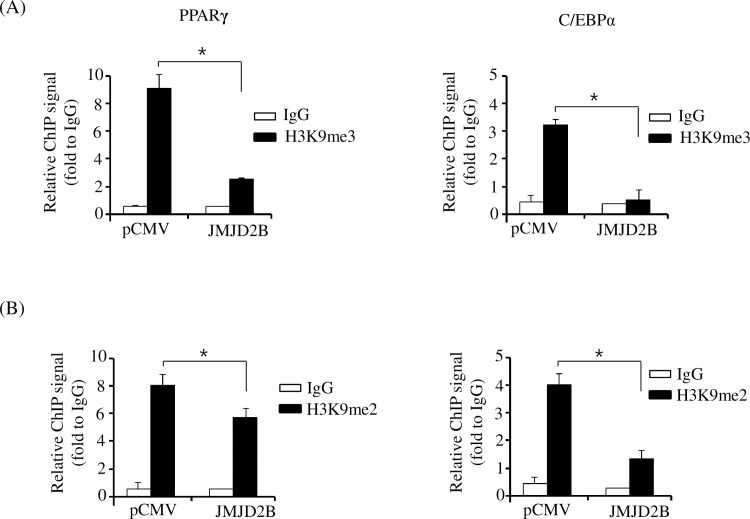
Overexpression of JMJD2B decreased the enrichment of H3K9me3 and H3K9me2 on the promoters of PPARγ and C/EBPα. The 3T3-L1 preadipocytes were transfected with JMJD2B expression vector and differentiated in the differentiation medium for four days. The enrichment of H3K9me3 (A) and H3K9me2 (B) was analyzed on the promoter of PPARγ and C/EBPα by ChIP-qPCR. Data are presented as means±SEM from three independent experiments. *P< 0.05 vs. control vector pCMV.

To further validate JMJD2B-mediated reduction of H3K9me3 and H3K9me2 enrichment during adipogenesis, we determined the enrichment of H3K9me3 and H3K9me2 on the promoter of PPARγ and C/EBPα in JMJD2B-overexpressed 3T3-L1 cells by ChIP-qPCR. The 3T3-L1 preadipocytes were transfected with the JMJD2B expression vector and stimulated to differentiate for four days. The ChIP-qPCR showed that the enrichment of H3K9me3 on the promoters of PPARγ and C/EBPα decreased markedly in JMJD2B overexpressed-3T3-L1 adipocytes at day 4 compared with the control cells (Fig 6A). The enrichment of H3K9me2 also efficiently decreased by JMJD2B overexpression (Fig 6B). These results suggest that JMJD2B decreases the enrichment of H3K9me3 and H3K9me2 on the promoters of PPARγ and C/EBPα.

### JMJD2B-mediated reduction of H3K9me3 increases H3K4me3 level on PPARγ and C/EBPα

**Fig 7:**
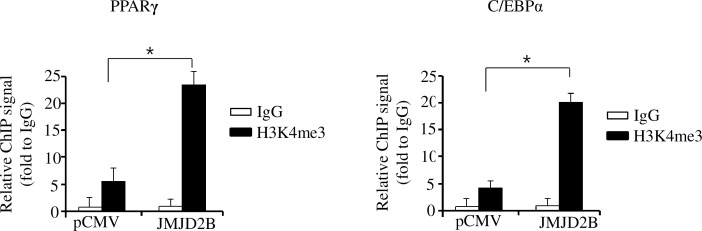
Decreased H3K9me3 favors the enrichment of H3K4me3 on the promoters of PPARγ and C/EBPα. The 3T3-L1 preadipocytes were transfected with the JMJD2B expression vector and differentiated in the differentiation medium for four days. The enrichment of H3K4me3 was analyzed on the promoter of PPARγ and C/EBPα by ChIP-qPCR. Data are presented as means±SEM from three independent experiments. *P< 0.05 vs. control vector pCMV.

Recently, it has been reported that active H3K4me3 and repressive H3K9me3 are mutually exclusive on the same nucleosome [17, 18]. In addition, H3K9me3 was associated with the repression of PPARγ and C/EBPα expression, whereas H3K4me3 was coordinated with the activation of PPARγ and C/EBPα expression [19, 20]. Therefore, we hypothesized that the methylation of H3K9me3 on PPARγ and C/EBPα promoters may prevent active H3K4me3 from occupying the promoters, and thus repress the expression of PPARγ and C/EBPα. Since we demonstrated in the present study that JMJD2B removes H3K9me3 on the promoters of PPARγ and C/EBPα, we investigated whether this JMJD2B-mediated removal of H3K9me3 favors the occupancy of H3K4me3 on the promoters. To this end, we determined the methylation status of H3K4me3 in JMJD2B-overexpressd 3T3-L1 cells at day 4 where H3K9me3 markedly decreased. The ChIP-qPCR analysis revealed that H3K4me3 significantly increased on the promoters of PPARγ and C/EBPα in JMJD2B-overexpressd 3T3-L1 cells at day 4 (Fig 7). These results suggest that demethylation of H3K9me3 induces sufficient H3K4me3 methylation on the promoters of PPARγ and C/EBPα, which may contribute to the stimulation of PPARγ and C/EBPα expression.

## Discussion

PPARγ and C/EBPα are considered the master regulators and stimulate adipocyte gene expression during adipogenesis. Therefore, they have been shown to play a pivotal role in terminal differentiation of preadipocyte [1, 2]. Several reports showed that the regulation of PPARγ and C/EBPα expression is accompanied by several histone modifications. In particular, methylation of histone H3K9me3 and H3K9me2 by histone methyltransferases such as SetdB1, Suv39h1, and G9a, was associated with the repression of PPARγ and C/EBPα expression and inhibition of adipogenesis [3–5]. Histone methylation by histone methyltransferases is reversed by histone demethylases. Therefore, the histone demethylase antagonizing H3K9me3 and H3K9me2 on PPARγ and C/EBPα may play an important role in the regulation of PPARγ and C/EBPα expression and adipogenesis. However, H3K9 demethylases that remove H3K9me3 and H3K9me2 on PPARγ and C/EBPα and promote adipogenesis have not yet been well characterized. In the present study, we demonstrated that JMJD2B is the histone demethylase responsible for removing H3K9me3 and H3K9me2 on the promoters of PPARγ2 and C/EBPα, and stimulates their expression during adipogenesis in 3T3-L1 preadipocytes.

A recent study has shown that JMJD2B acted as a cofactor of C/EBPβ, demethylated H3K9me3 on C/EBPβ target genes, and promoted mitotic clonal expansion, resulting in stimulation of adipogenesis [15]. However, it is not known whether JMJD2B demethylates H3K9me3 and H3K9me2 on PPARγ and C/EBPα. Since JMJD2B is a histone demethylase that reverses H3K9me3/me2, we investigated whether JMJD2B positively regulates expression of PPARγ and C/EBPα by affecting H3K9me3/me2 on their promoters. JMJD2B knockdown using siRNA decreased PPARγ and C/EBPα expression, whereas significantly increased H3K9me3 and H3K9me2 on the promoters of PPARγ and C/EBPα, suggesting that the repression of PPARγ and C/EBPα by JMJD2B knockdown is coordinated with increased enrichment of H3K9m3/me2 on PPARγ and C/EBPα. We also validated the role of JMJD2B on the regulation of the enrichment of H3K9me3/me2 on the promoters of PPARγ and C/EBPα in JMJD2B-overexpressed 3T3-L1 adipocytes four days after differentiation. In JMJD2B overexpressed-3T3-L1 adipocyte cells at four days after differentiation, PPARγ and C/EBPα expression increased, but the enrichment of H3K9me3/me2 on the promoters of PPARγ and C/EBPα, decreased markedly compared with the control, indicating that overexpression of JMJD2B reduces H3K9me3/me2 on the promoters of PPARγ and C/EBPα and thus stimulates the expression of PPARγ and C/EBPα. A previous study has shown that LSD removed H3K9me2 on C/EBPα and increased C/EBPα expression, which caused to stimulation of adipogenesis [15]. Unlike LSD, our study showed that JMJD2B can demethylate both H3K9me3 and H3K9me2 on the promoters of PPARγ and C/EBPα and then positively regulate the expression of PPARγ and C/EBPα. These results suggest that JMJD2B may contribute to the stimulation of terminal adipocyte differentiation by activating PPARγ and C/EBPα expression through the removal of H3K9me3 and H3K9me2 on their promoters. Therefore, JMJD2B may play a positive role in terminal adipocyte differentiation as well as early adipocyte differentiation through stimulation of mitotic clonal expansion.

Recently, it has been reported that the demethylation of H3K9 by JMJD2B might be a necessary precondition for H3K4 methylation by the mixed-lineage leukemia (MLL) 2 complex in coactivating estrogen receptor α (ERα)-mediated transcription [18]. In the same study, it has been shown that JMJD2B and the MLL2 complex interact to define the methylation state of H3K4 and H3K9 in ERα-activated transcription, in which H3K9 demethylation precedes H3K4 methylation. Their findings clearly demonstrate that H3K9 demethylation is required for sufficient H3K4 methylation. In addition, several reports have shown that the coordinated methylation of H3K9 and H3K4 is involved in the regulation of adipogenesis [3–5, 19, 20]. Tri-methylation of H3K9 by Suv39H1 and Setdb1 was associated with repression of PPARγ and C/EBPα, while tri-methylation of H3K4me3 by MLL4, a H3K4me3 methyltransferase and methylation regulator PTIP, was associated with activation of PPARγ and C/EBPα [19, 20]. Therefore, we hypothesized that the demethylation of H3K9me3 induced by JMJD2B may affect the enrichment of H3K4me3 on PPARγ and C/EBPα during adipogenesis in 3T3-L1 preadipocytes. To test our hypothesis, we determined the enrichment of H3K4me3 on PPARγ and C/EBPα in JMJD2B-overexpressed 3T3-L1 adipocyte cells because JMJD2B overexpression reduces the enrichment of H3K9me3 on the promoters of PPARγ and C/EBPα. The enrichment of H3K4me3 on PPARγ and C/EBPα markedly increased in JMJD2B-overexpressed 3T3-L1 adipocytes, in which the expression levels of PPARγ and C/EBPα increased. These results indicate that JMJD2B-mediated demethylation of H3K9me3 might be a necessary precondition for methylation of active H3K4me3 on PPARγ and C/EBPα by the MLL4 complex and activation of PPARγ and C/EBPα expression.

In conclusion, we have demonstrated that JMJD2B is the histone H3K9 demethylase responsible for the demethylation of H3K9me3/me2 on the promoters of PPARγ and C/EBPα and positively regulates their expression. Therefore, JMJD2B may play an important role in terminal differentiation of 3T3-L1 cells.

## Supporting Information

S1 Table List of primers for qPCR. (DOC)
