# Supplementary material for: Histone H3K9 Demethylase JMJD2B Activates Adipogenesis by Regulating H3K9 Methylation on PPARγ and C/EBPα during Adipogenesis
Source: PLoS One. 2017 Jan 6;12(1):e0168185. doi: 10.1371/journal.pone.0168185 (PMC5218499; doi:10.1371/journal.pone.0168185)
Supplement: S1 Table — (DOC) [file pone.0168185.s001.doc]

S1 Table List of primers for q- PCR

| Gene | Forward primer | Reverse primer |
| --- | --- | --- |
| JMJD2B | GGCCAAGATCATTCCACCCA | CCCACAGTCATGGCCTTCTT |
| PPARγ | GTGCCAGTTTCGATCCGTAGA | GGCCAGCATCGTGTAGATGA |
| C/EBPα | CAAGAACAGCAACGAGTACCG | GTCACTGGTCAACTCCAGCA |
| C/EBPβ | AAGCTGAGCGACGACGAGTACAAGA | GTCAGCTCCAGCACCTTGTG |
| aP2 | ACACCGAGATTTCCTTCAAACTG | CCATCTAGGGTTATGATGCTCTTC |
| m18S | CGGCTACCACATCCAAGGAA | GCTGGAATTACCGCGGCT |
